# Artificial Intelligence Algorithm Based on Genetics to Predict Responses to Interferon-Beta Treatment in Multiple Sclerosis Patients

**DOI:** 10.3390/bioengineering13050523

**Published:** 2026-04-30

**Authors:** Edgar Rafael Ponce de León-Sánchez, Jorge Domingo Mendiola-Santibañez, Omar Arturo Domínguez-Ramírez, Ana Marcela Herrera-Navarro, Alberto Vázquez-Cervantes, Hugo Jiménez-Hernández, José Alfredo Acuña-García, Rafael Duarte-Pérez, José Manuel Álvarez-Alvarado

**Affiliations:** 1 Facultad de Informática, Universidad Autónoma de Querétaro, Querétaro 76230, Mexico; mherrera@uaq.mx (A.M.H.-N.); hugo.jimenez@uaq.edu.mx (H.J.-H.); jose.alfredo.acuna@uaq.mx (J.A.A.-G.); rafael.duarte@uaq.mx (R.D.-P.); 2 Facultad de Ingeniería, Universidad Autónoma de Querétaro, Querétaro 76010, Mexico; mendijor@uaq.mx (J.D.M.-S.); jmalvarez@uaq.edu.mx (J.M.Á.-A.); 3 Centro de Investigación en Tecnologías de Información y Sistemas, Universidad Autónoma del Estado de Hidalgo, Pachuca 42039, Mexico; 4 Centro de Ingeniería y Desarrollo Industrial, Querétaro 76125, Mexico; alberto.vazquez@cidesi.edu.mx

**Keywords:** multiple sclerosis, machine learning, biomarkers, fuzzy system, genetic algorithm

## Abstract

Multiple sclerosis (MS) is an inflammatory disease of the central nervous system (CNS) that impacts nearly 3 million people worldwide. While the etiology and pathogenesis of MS are not yet fully understood, current evidence suggests that it results from complex interactions between genetic and environmental conditions. Clarifying the autoimmune mechanisms underlying MS remains a central objective in the development of effective therapeutic strategies. Interferon-beta (IFN-β) is one of the most frequently prescribed disease-modifying treatments for individuals with MS. However, despite its established efficacy, recent studies report that approximately 30–50% of patients exhibit inadequate response to IFN-β, largely due to genetic variability. Machine learning (ML), a branch of artificial intelligence (AI), employs data-driven computational models to enhance predictive accuracy and classification. In recent MS research, unsupervised learning techniques such as hierarchical clustering and K-means have been applied for classification purposes. However, these methods often fail to yield optimal solutions because they require numerous arbitrary decisions and perform adequately only when datasets contain clusters of similar sizes and lack significant outliers. Fuzzy systems (FSs) are designed to model complex, ambiguous real-world phenomena. In this study, an AI algorithm incorporating a fuzzy system, informed by expert neurologist input, is proposed to enhance the assignment of unknown class labels related to IFN-β response in MS patients. Additionally, a genetic algorithm (GA) is introduced to identify optimal solutions within the search space, facilitating hyperparameter optimization of a deep learning (DL) model trained with genetic biomarkers to identify patients likely to benefit from this therapy. Experimental results demonstrate that the fuzzy system achieved 80% classification efficiency, in contrast to 64% with conventional hierarchical clustering. Furthermore, an artificial neural network (ANN) model, with hyperparameters optimized by the GA, achieved an accuracy of 0.8–1.0, surpassing the multi-layer perceptron (MLP), which achieved 0.6–0.8 accuracy using conventional tuning methods.

## 1. Introduction

Multiple sclerosis (MS) is an autoimmune inflammatory disease that affects the central nervous system (CNS) [1]. MS is pathologically identified by some degrees of demyelination, axonal loss, and gliosis and clinically by a diversity of neurological symptoms [2]. Usually, the most common initial symptoms are sensory disturbances, motor alterations, and brainstem dysfunction [3]. There are three subtypes of MS; the most prevalent is relapsing-remitting multiple sclerosis (RRMS), described by alternating periods of remission and symptom exacerbation. In contrast, primary progressive multiple sclerosis (PPMS) is distinguished by a continuous progression of symptoms without remission. Secondary progressive multiple sclerosis (SPMS) begins as RRMS, and the next phase is characterized by a progressive decrease in neurological function [4]. The etiology and pathogenesis of MS are not clearly determined but are likely the result of complex relations between genetic and environmental conditions [5]. Environmental conditions such as viral infections, vitamin D deficiency, and smoking activate the human immune system, leading to immune dysregulation and an immune attack on the myelin sheaths of the CNS [6,7,8]. Immune dysregulation manifests as infiltration of immune cells, which is influenced by specific genetic factors that remain largely unknown.

MS treatment is complex and involves agents that act through various mechanisms. Therapeutic strategies are determined by the clinical course and the specific subtype of the disease. MS treatment can be categorized into three types: symptom treatment, relapse treatment, and disease-modifying therapy. The primary objective of MS treatment is to slow disease progression. Glatiramer acetate and interferon beta (IFN-β) are widely recognized as first-line therapies for MS. For patients who exhibit inadequate response to these therapies, several second-line therapies are available, such as natalizumab and fingolimod [9]. IFN-β is one of the most frequently prescribed disease-modifying treatments for RRMS patients. IFN-β has many ways of affecting the immune system: It can prevent activated T-cell production, prevent the migration of activated immune cells across the blood-brain barrier (BBB), inhibit the proliferation of pro-inflammatory cytokines (e.g., IL-2, IL-12, and IFN-γ), and lead to an increase in anti-inflammatory cytokines (e.g., IL-4, IL-5, IL-10, and TGF-β). Additionally, IFN-β contributes to remyelination within the CNS [10,11]. IFN-β also prevents the differentiation of Th1/Th17 inflammatory cells and shifts the Th cell phenotype from Th1 inflammatory to Th2 anti-inflammatory [12]. Recently, Li et al. [13] conducted a multiplex analysis of short-term and long-term IFN-β responses in patients with relapsing–remitting multiple sclerosis (RRMS). In untreated multiple sclerosis (MS) patients, Th1, Th2, and neurotrophic protein levels were subnormal and exhibited weak intercorrelations. Long-term IFN-β treatment increased serum protein levels and restored a balanced, positively correlated profile, comparable to that observed in healthy controls. Previous studies have analyzed the IFN-β treatment effects on cytokine patterns in RRMS patients [14,15]. Other investigations have demonstrate that IFN-β reduces disease activity [16,17]. The current literature suggests that, despite the efficacy of IFN-β, approximately 30–50% of patients do not respond to it due to individual genetic variability [18].

## 2. Background

Technological advances in recent decades have promoted large-scale studies of genes, proteins, and metabolites, resulting in the emergence of omics disciplines like genomics, proteomics, and metabolomics. These fields have significantly improved the understanding of disease etiology. In genomics, the analysis of large-scale data has become more efficient, a process that was previously challenging with conventional statistical techniques [19]. The human genome comprises approximately 20,000 genes, and the complexity and multidimensionality of this data require the application of artificial intelligence (AI) methods, including expert systems, machine learning (ML), deep learning (DL), and evolutionary algorithms, to enable more efficient data processing [20].

To date, the field of expert systems has been one of the most active in different research areas, such as disease diagnosis. An expert system is a computer scheme that emulates the reasoning procedure of a human expert and can efficiently manage uncertain and imprecise information [21]. A fuzzy expert system includes fuzzy sets and fuzzy logic in its reasoning procedure and knowledge base. Some studies have applied fuzzy systems (FSs) to analyze neurological diseases [22]. However, most of these studies have only focused on MS diagnosis [23,24,25,26]. The study of responses to MS treatments remains a challenge.

ML and DL algorithms are based on mathematical models that search complex patterns in data and are arising as useful tools in bioinformatics [27,28,29]. Over the past decade, the application of ML algorithms has increased across different medical fields, including cardiology, oncology, and neurology [30,31]. ML is classified into two types: supervised learning, which trains a model with known inputs and outputs to predict future outcomes, and unsupervised learning, which identifies hidden patterns in data without labeled outputs. These learning models, trained with genetic features of patients, can help improve the diagnosis of some diseases, such as early MS [32,33,34], and estimate the potential response to MS treatments, such as natalizumab and fingolimod [35,36].

Hierarchical clustering is an unsupervised learning algorithm that groups data into a tree of nested sets. Several studies have utilized hierarchical and non-hierarchical clustering methods to classify treatment responses in MS patients [37,38]. Additional studies have analyzed genomic data to search for genetic factors associated with the response to IFN-β therapy. For example, Gurevich et al. [39] classified 50 SPMS patients (20 treated with IFN-β-1a and 30 untreated) using hierarchical clustering based on the IFN-inducible gene expression profile identified in clinically responsive RRMS patients. The gene expression profile was previously determined by searching for differentially expressed genes (DEGs) among IFN-treated (*n* = 10) and untreated (n = 25) RRMS patients. As a result, 104 DEGs, enriched by the IFN signaling way (*p* = $7.4\times 10^{8}$), were identified in RRMS patients treated with IFN. In ref. [40], Coelewij et al. identified early immunogenicity biomarkers associated with the future development of antidrug antibodies (ADAs) in RRMS patients before their first IFN-β treatment. DEGs in SPMS patients versus RRMS ADAneg and ADApos versus RRMS ADAneg patients were used to classify patients using hierarchical clustering and principal component analysis (PCA). The results indicated that these biomarkers could aid future treatment decisions in RRMS patients.

K-means is an unsupervised iterative clustering algorithm that employs centroid calculations to divide a dataset into similar clusters using the distance between their centroids. Some studies have implemented this method for classification tasks. Mesidor et al. [41] proposed a group-based trajectory modeling (GBTM) approach to identifying patient groups with homogeneous dosing patterns for the drugs IFN-β-1a and amitriptyline. Characteristics differences between clusters were identified employing chi-square and analysis of variance, both weighted by the latter probability of cluster membership. As a result, seven dosing patterns were identified for IFN-β-1a, and five were identified for amitriptyline. In contrast, Kular et al. [42] analyzed deoxyribonucleic acid (DNA) methylation in the core of non-neuronal cells separated from 38 samples of normal-appearing white matter (NAWM) from MS patients, contrasted to control individuals. A total of 1226 differentially methylated positions (DMPs) with adjusted *p*-values across the genome (*p*-adj < 0.05) were identified between MS patients and healthy controls. Genes involved in biological processes were classified using K-mean clustering into five groups. The findings suggest that NAWM glial cells are highly altered, revealing relevant molecular changes underlying MS neuropathology. Although unsupervised learning techniques are relatively easy to implement, some limitations are identified: (1) Hierarchical clustering rarely provides the best solution because of the wide number of arbitrary decisions. (2) K-means works effectively only when the dataset includes groups of similar size, and there are no notable outliers.

Tao et al. [43] implemented a feature selection method to identify differentially correlated gene (DCE) pairs associated with IFN-β-1b treatment response in RRMS patients. As a result, 22 statistically significant DCE (*p* < 0.01) were identified, involving 41 unique genes. Utilizing these biomarkers as predictors, a support vector machine (SVM) model was trained, achieving a predictive accuracy of 0.8095. However, some limitations are apparent: (1) The training dataset does not contain output labels, and the method used to determine the IFN-β-1b responder and non-responder categories is not clearly described. (2) The training dataset only has 25 samples in total, and to delete potentially ambiguous results caused by patients whose first relapse occurred after 5 years, 7 patients were excluded from the analysis, resulting in a loss of potentially relevant information. (3) Parameter-based learning models, such as the SVM model, are trained by tuning their hyperparameters. These are configurations that are usually tuned arbitrarily before the training process begins to optimize model performance. However, arbitrary hyperparameter tuning does not always yield the best performance [44].

In contrast, genetic algorithms (GAs) are metaheuristic methods that search for optimal solutions in the search space and can be useful for optimizing the hyperparameters of learning models to achieve higher performance [45]. Therefore, this study attempts to address some limitations of previous studies and proposes an AI algorithm that includes following:

- A method for validating the normal distribution of a dataset containing demographic, clinical, and genetic variables from 25 RRMS patients treated with IFN-β-1b for two years.
- A fuzzy expert system, based on the opinion of a neurology expert, to improve the efficiency of assigning unknown class labels from the dataset, using demographic and clinical features as input variables.
- A GA to optimize the configuration hyperparameter tuning of an artificial neural network (ANN) that is a DL model trained with genetic variables associated with the response to IFN-β-1b, with the goal of estimating whether new patients are potential aspirants to be treated with this therapy and achieving higher predictive performance than previous studies.

The clinical implementation of this algorithm could support specialists’ decision-making in estimating the response to MS treatments, preventing ineffective therapies, and reducing healthcare costs.

## 3. Materials and Methods

**Figure 1:**
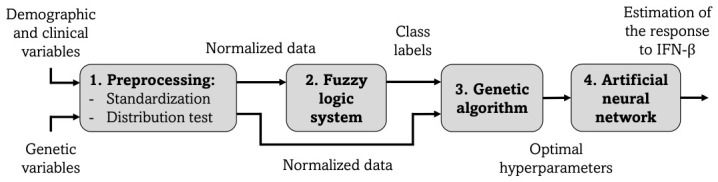
Proposed methodology. Demographic, clinical, and genetic features are imported from the database. Next, they are preprocessed using standardization and distribution test techniques. Then, the demographic and clinical variables are fed into a fuzzy logic system to obtain unknown class labels. Finally, the genetic variables with their labeled outputs are fed into a GA to find the optimal hyperparameter configuration for training an ANN model to perform predictions.

The implemented research strategy is described in the flowchart shown in Figure 1.

### 3.1. Database

The training and validation datasets were collected from the GSE24427 array expression profiling experiment, available in the Gene Expression Omnibus (GEO) repository (https://www.ncbi.nlm.nih.gov/geo/query/acc.cgi?acc=GSE24427) (accessed on 15 October 2025). The database is the same as that used by the authors of [43], although the selected biomarkers differ. The GSE24427 experiment is described as follows:

- Experiment Type: Expression profiling by array.
- Platform: GPL96 [HG-U133A] Affymetrix Human Genome U133A Array.
- Expression Data: Genome-wide expression profiles of peripheral blood mononuclear cells from 25 German MS patients.
- MS Type: Relapsing–remitting.
- Therapy: Subcutaneous IFN-β-1b (Bayer Healthcare).
- IFN-β-1b Dose: Betaferon, 250 μg every other day.
- Blood Sampling Period: Before the first and second injections and at months 1, 12, and 24 of IFN-β-1b injection.

Expression data were imported using GEO2R, a web tool that enables visualization of specific gene expression values through the Profile Graph function. Additional information about the GEO2R options feature is available at https://www.ncbi.nlm.nih.gov/geo/info/geo2r.html#options_feature. The GPL96 platform provides demographic and clinical features of RRMS patients, which are presented in Table 1.

**Figure 2:**
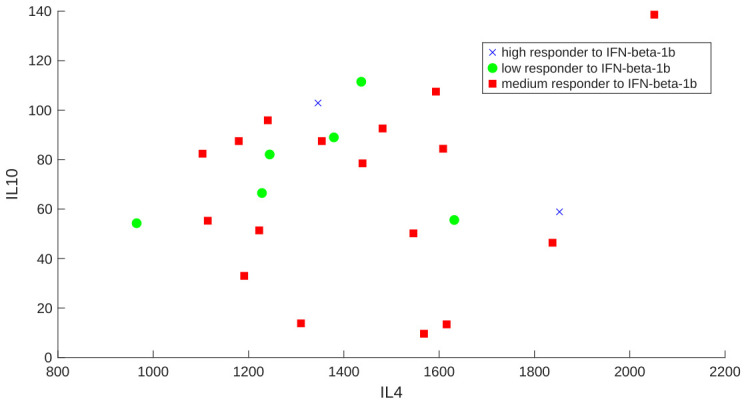
Paired genetic expression values of IL4/IL10 cytokines in 25 RRMS patients before the first dose of IFN-β-1b. The ‘responder to IFN-β-1b’ classification is based on the opinion of an expert neurologist.

In addition, the expression values of 13 genetic biomarkers (IL-2, IFN-γ, TNF-α, IL-4, IL-10, TGF-β, CD46, CD58, FHIT, IRF5, GAPVD1, GRM3, and GRIK2) were imported to train the proposed ANN model. These genetic biomarkers were selected because, in previous studies [12,14,15,16,17], the authors observed that the response to IFN-β treatment in MS patients is associated with their expression levels. For example, the expression values of the IL4 and IL10 cytokines are shown in Figure 2.

### 3.2. Data Standardization

To preprocess the input database, standardization is first applied to normalize the demographic, clinical, and genetic variables. This method normalizes the characteristics by eliminating the mean μ, and it scales them by dividing by their standard deviation *s* [34]. The standard score *z* of a sample *x* is

(1) $$z=\frac{x-\mu }{s}.$$

### 3.3. Data Distribution Test

Prior to entering standardized data into the fuzzy system and GA, it is important to evaluate whether the input variables exhibit a normal distribution [46]. The Kolmogorov–Smirnov (KS) test, a nonparametric method, determines if a dataset conforms to a specified distribution. The KS test contrasts the observed cumulative distribution function of a variable with a determined theoretical distribution, such as normal, uniform, Poisson, or exponential distribution. The test statistic represents the maximum absolute difference between the noticed and theoretical cumulative distribution functions. This test evaluates whether the sample data are consistent with the determined distribution. Equation (2) describes the empirical independent distribution function $F_{n}$ for *n*-ordered, and identically distributed observations, $X_{i}$:

(2) $$F_{n}\left(x\right)=\frac{1}{n}\sum_{i=1}^{n}1_{\left(-\infty ,x\right]}X_{i}$$

where $1_{\left(-\infty ,x\right]}X_{i}$ is the indicator function, which is the same as 1 if $X_{i}\leq x$, or it is the same as zero otherwise.

The KS statistic for a given cumulative distribution function F(x) is

(3) $$D_{n}=sup_{x}\left|F_{n}\left(x\right)-F\left(x\right)\right|$$

where $sup_{x}$ is the supremum of the set of distances. The statistic represents the biggest absolute difference between the two distribution functions through all values of *x*. The KS test is also applicable to determine whether two underlying one-dimensional probability distributions differ. In this context, the KS statistic is

(4) $$D_{n,m}=sup_{x}\left|F_{1,n}\left(x\right)-F_{2,m}\left(x\right)\right|$$

where $F_{1,n}\left(x\right)$ and $F_{2,m}\left(x\right)$ are the empirical distribution functions of the first and second samples, respectively.

### 3.4. Assignment of Class Labels

Since the training dataset does not contain output information, a modified fuzzy system similar to that proposed in [47] is used to assign the unknown class labels, i.e., to classify RRMS patients as high, medium, and low responders to IFN-β-1b treatment, based on their demographic and clinical characteristics. Additionally, a hierarchical clustering algorithm is trained to classify the same patients for comparison purposes.

#### 3.4.1. Fuzzy Logic System

The main motivation for introducing fuzzy set theory was to model real-world phenomena characterized by ambiguity and limited observability. Human understanding of complex problems can be effectively represented using imprecise natural language terms [48]. Fuzzy systems are schemes based on fuzzy set theory and fuzzy logic designed to efficiently process imprecise data [49]. Their principal attribute is the symbolic description of knowledge as fuzzy conditional rules. The standard scheme of a fuzzy system consists of three stages: fuzzifier, approximate reasoning, and defuzzifier.

**Figure 3:**
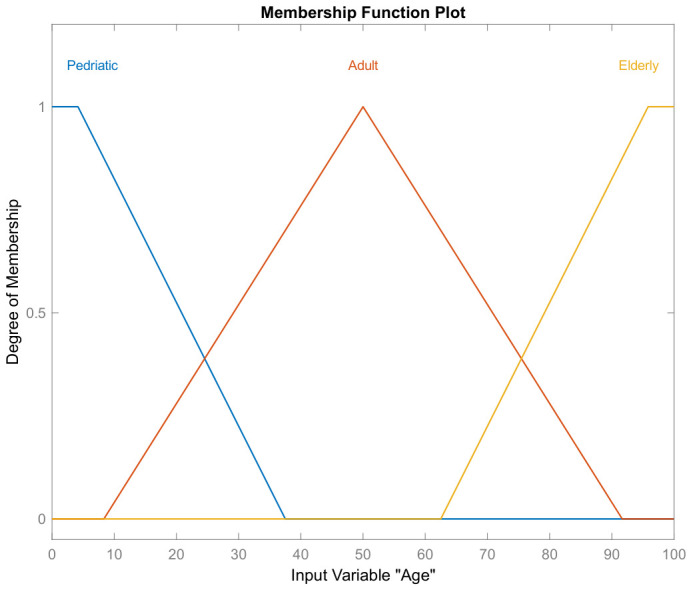
Set of linguistic values regarding three labels representing the input variable ‘Age’ (fuzzy set $A_{1}$).

**Figure 4:**
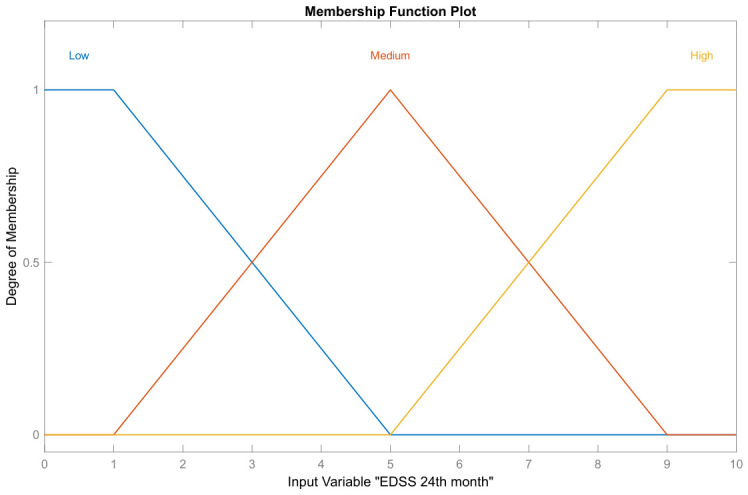
Set of linguistic values regarding three labels representing the input variable ‘EDSS after two years’ (fuzzy set $A_{4}$).

The fuzzifier converts the values of the input variable into a fuzzy set *A* of linguistic values using approximate reasoning (inference engine) and expert knowledge described as a set of fuzzy conditional rules (knowledge base). The fuzzifier is described as the membership function $\mu_{A}\left(x\right)$ of the fuzzy set *A*. The demographic and clinical features of RRMS patients are employed as input variables for the fuzzifier. The approximate reasoning result is a fuzzy set B(y). The defuzzifier calculates an average numerical output from the fuzzy set B(y) result. The numerical output $y_{0}$ is calculated via the center of gravity (COG) method:

(5) $$y_{0}=\frac{\sum_{i=1}^{n}y_{i}\mu_{B}\left(y_{i}\right)}{\sum_{i=1}^{n}\mu_{B}\left(y_{i}\right)},$$

where $\mu_{B}\left(y\right)$ describes the membership function of the fuzzy set B(y) [50]. For the implementation of the fuzzy system, the input linguistic variables are described in Table 2. The sets of possible linguistic values are groups of different labels representing the inputs: age, initial EDSS, EDSS after one year, EDSS after two years, and the output: response to IFN-β-1b. For example, the graphs of the membership functions $\mu_{A_{1}}$(age) and $\mu_{A_{4}}$(EDSS after two years) of the fuzzy sets $A_{1}$ and $A_{4}$, respectively, are shown in Figure 3 and Figure 4.

The Fuzzy Logic Designer application (MATLAB R2023a) is employed to implement the fuzzy system. Its structure is based on the Mamdani-Assilian Fuzzy Logic System (MAFS) [51], which composes a set of fuzzy rules. Table 3 shows some of the 33 conditional rules based on the opinion of a neurology expert. The complete fuzzy rules are presented in Table A1 and Table A2.

#### 3.4.2. Hierarchical Clustering

Hierarchical clustering refers to a family of unsupervised machine learning algorithms that construct nested groups by successively dividing them. The resulting clustering hierarchy is typically described as a tree structure, known as a dendrogram. The root of the tree corresponds to a single cluster involving all samples, while the leaves describe clusters involving individual samples. These algorithms generally employ a bottom-up approach, where each sample initially forms its own cluster and clusters are repeatedly combined. The linking criteria specify the metric utilized for the combining method [52]. Hierarchical clustering can group a large number of samples when implemented with a connectivity matrix; however, it becomes computationally costly without connectivity restrictions, as all possible divisions must be considered at each step.

### 3.5. ML and DL Algorithms

Once the unknown class labels of the dataset have been assigned, a classical ML model, such as the multi-layer perceptron (MLP) [53], and an artificial neural network (ANN) model [54] are trained with the genetic features of 25 RRMS patients to estimate the likely response to IFN-β-1b and compare the performance of the learning models. For this purpose, the software Anaconda3 2021.05 and the open-source web application Jupyter Notebook 6.3.0 are employed to create the pseudocode that runs on a Lenovo 11th Gen Intel(R) Core(TM) i5-1135G7 2.40GHz 64 bits, manufactured in Beijing, China. The versions of the Python 3.8.8 software libraries are Keras 2.9.0 (TensorFlow) and Sklearn 1.3.2 (Python).

Deep learning (DL) models have shown major performance improvements over traditional machine learning (ML) models [55,56,57,58] because they can learn highly complex connections between input and output data. ANN models form the foundation of DL and are mathematical constructs able to describe complex functions as compositions of simpler functions. The fundamental unit of these functions is the neuron, which executes a linear conversion of the input, such as multiplying the input by a weight and adding a bias, followed by the application of a fixed nonlinear or activation function [59].

Mathematically, the output can be written like o=f(w×x+b), where *f* is the activation function, *w* is the weight, *x* is the input, and *b* is the bias. The previous expression is known as a layer of neurons, as it describes many neurons through multidimensional weights and biases. When the number of such layers exceeds two, the neural system is called a deep neural network. A deep or multilayer neural network is a composition of nonlinear functions,

(6) $$x_{1}=f\left(w_{0}\times \left(x+b_{0}\right)\right)$$

(7) $$x_{2}=f\left(w_{1}\times \left(x_{1}+b_{1}\right)\right)$$

(8) ⋮

(9) $$y=f(w_{n}\times \left(x_{n}+b_{n}\right))$$

where the output of one layer of neurons is utilized as the input for the next layer, $w_{0}$ is a matrix, and *x* is a vector. Employing a vector lets $w_{0}$ contain a whole layer of neurons and not only a single weight.

The genetic input data used to train the proposed ANN model are divided into 80% for training ($X_{train}$) and 20% for validation ($X_{test}$) according to Pareto analysis [60]. Furthermore, the output labels are equally divided into 80% for training ($y_{train}$) and 20% for validation ($y_{test}$).

Overfitting is a common phenomenon in ML and DL, where a model performs well with training data but not with validation data, i.e., the model is too complex with high variance. On the other hand, a model can have underfitting (high bias) if the model is too easy. To obtain a balanced variance-bias rate, the k-iteration cross-validation (CV) method is implemented. CV randomly divides the training dataset into *k* iterations without substitution, where k−1 iterations are employed for training, and one iteration for performance testing. This operation is repeated *k* times or folds to obtain *k* models and to achieve satisfactory generalization performance [53].

Furthermore, it is important to evaluate the predictive performance of the learning models by computing some commonly used metrics: the confusion matrix (CM), accuracy, precision, and sensitivity (recall). Moreover, categorical cross-entropy ($L(y,\hat{y})$) is the loss function used to measure the performance of the ANN model. This function calculates the contrast between the true labels $y_{i}$ for class *i* and the predicted probabilities $\hat{y}$ for class *i*,

(10) $$L\left(y,\hat{y}\right)=-\sum_{i=1}^{C}y_{i}\;log\left({\hat{y}}_{i}\right)$$

where *C* denotes the number of classes.

### 3.6. Hyperparameter Optimization via GA

Similar to any other hyperparameter-based ML model, a DL model is trained by tuning its hyperparameters. To train a neural network, an optimization scheme must be adopted. Hyperparameters are configurations of a learning model that can be randomly tuned before beginning the training procedure to optimize the model’s performance. In contrast, model parameters, like neural network weights *w*, are learned during training [61].

**Figure 5:**
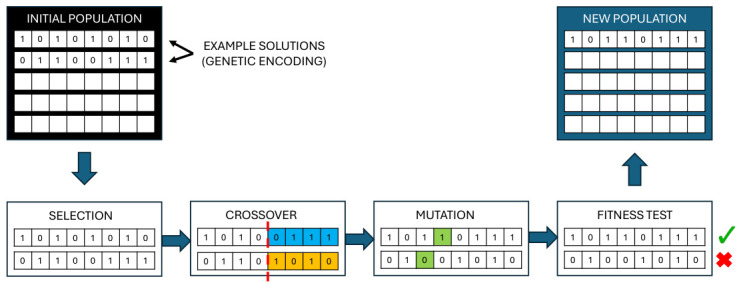
The The initial population is randomly selected from the search space, and its members (example solutions) are encoded as chromosomes represented as bit strings (black arrows). The chromosome is composed of genes. The values that genes take are alleles, which can be binary over the binary alphabet {0,1}. The genetic operations of selection, crossover, and mutation are repeatedly applied to the population until the end criterion is met. At the end of each iteration, the population’s fitness values are computed. The members with the highest fitness (checkmark in green) are selected for evolving and reproduction (new population). The members with the lowest fitness (crossmark in red) are discarded. Finally, the member with the highest fitness value is the optimal solution to the problem.

In this study, a genetic algorithm (GA) is proposed to find the optimal hyperparameters defining the architecture of an artificial neural network (ANN) trained with genetic variables associated with the response to IFN-β-1b. GA is an iterative searching technique based on populations that merges natural selection and genetics. GA seeks the optimal solution in the search space [62]. GA converges in a finite number of iterations (generations), and contender solutions evolve toward greater fitness or quality in each iteration. The algorithm ends when an optimal solution, a maximum number of iterations, or a stopping condition is reached. The GA stages involve initial population selection, a target or fitness function, genetic operators, and ending criteria [63]. The algorithm begins by describing the problem and its target function f(X), where *X* is a multidimensional vector. Figure 5 shows the schematic diagram of how a GA works.

Genetic operators integrate the principal search mechanisms of GA and are used to create new solutions from existing population members. The two basic types of operators are crossover and mutation. Crossover joins two individuals to generate two offspring, since mutation alters a single individual to produce a new solution. For real m-dimensional vectors $\bar{X}$ and $\bar{Y}$, which define chromosomes, the operators considered are uniform mutation and simple crossover. For each variable *i*, let $a_{i}$ and $b_{i}$ describe the lower and upper bounds, respectively. Uniform mutation includes randomly selecting a variable *j* and assigning it a value uniformly drawn between $a_{i}$ and $b_{i}$.

(11) $$x_{i}^{\prime }=\left\{\begin{array}{c} U(a_{i},b_{i})\;\;\;si\;i=j\\ x_{i}\;\;\;\;\;\;otherwise. \end{array}\right\}$$

The simple real-valued crossover creates a random number *r* from a uniform distribution and generates two new individuals ($X^{\prime }$ and $Y^{\prime }$) corresponding to Equation (12).

(12) $$x_{i}^{\prime }\left(y_{i}^{\prime }\right)=\left\{\begin{array}{c} x_{i}\left(y_{i}\right)\;\;\;\;\;\;si\;i<r\\ y_{i}\left(x_{i}\right)\;\;\;\;\;\;otherwise. \end{array}\right\}$$

The proposed GA starts by defining the problem (optimal hyperparameters) and its target function f(X) (maximum validation accuracy). In this case, *X* represents the following hyperparameter network options:

1. num_hidden_neurons: [64, 128, 256, 512, 768, 1024].
2. num_hidden_layers: [1, 2, 3, 4].
3. activation_function: [‘relu’, ‘elu’, ‘tanh’, ‘sigmoid’].
4. optimizer: [‘rmsprop’, ‘adam’, ‘sgd’, ‘adagrad’, ‘adadelta’, ‘adamax’, ‘nadam’].

Table 4 presents the pseudocode for the GA implementation. The chromosome (chosen hyperparameters) with the highest fitness represents the global optimal solution. For comparison purposes, the hyperparameter tuning of an MLP model (trained with the same genetic features) is also performed using the conventional GridSearchCV tool from Python’s sklearn library, which executes an exhaustive search for specific hyperparameter values for an estimator, considering all combinations. The hyperparameter network options for the MLP model are as follows:

1. num_hidden_neurons: [64, 128, 256, 512, 768, 1024].
2. num_hidden_layers: [1, 2, 3, 4].
3. activation_function: [‘identity’, ‘logistic’, ‘tanh’, ‘relu’].
4. solver: [‘lbfgs’, ‘sgd’, ‘adam’].

## 4. Results

In this study, demographic, clinical, and genetic characteristics of 25 RRMS patients treated with IFN-β-1b for two years were collected. Then, they were preprocessed using the standardization technique and the KS normal distribution test. Next, a fuzzy system was used to assign unknown class labels: high, medium, or low response to IFN-β-1b. Moreover, a hierarchical clustering model was implemented to classify the same patients. Subsequently, a GA was used to find the optimal configuration hyperparameter of an ANN model trained with the genetic variables associated with the labeled outputs. Moreover, an MLP model configured by a conventional hyperparameter tuning technique was trained with the same genetic features.

### 4.1. Data Distribution Test

**Figure 6:**
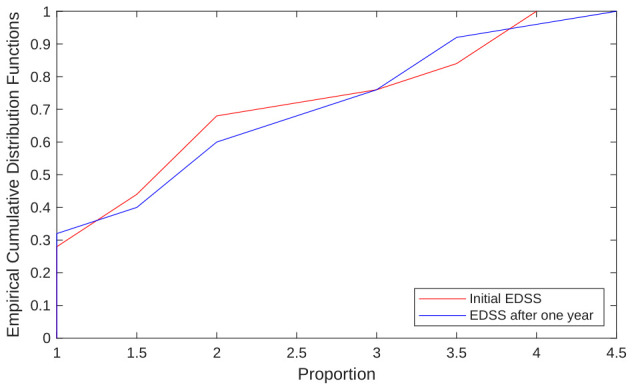
Graph of the empirical cumulative distribution functions of the clinical variables: initial EDSS and EDSS after a year. Both variables follow a normal distribution (*p*-value > 0.05).

After standardizing the demographic and clinical variables and applying the KS test to verify their normal distribution (*p*-value > 0.05), the results in Table 5 were obtained. For example, Figure 6 shows the empirical cumulative distribution functions of the ‘Initial EDSS’ and ‘EDSS after one year’ clinical variables.

**Figure 7:**
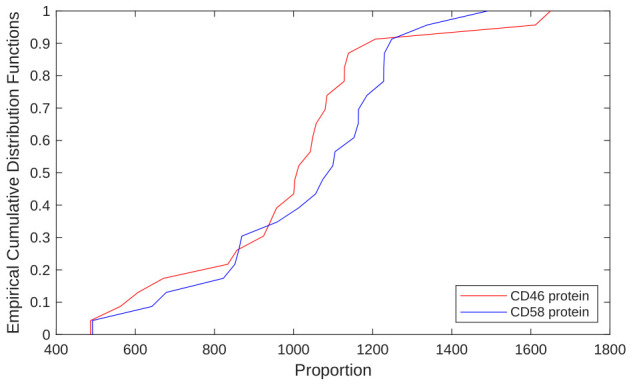
Graph of the empirical cumulative distribution functions of CD46 and CD58 proteins before the first dose of IFNβ-1b; both biomarkers follow a normal distribution (*p*-value > 0.05).

The genetic variables were also standardized, and after applying the KS test to validate their normal distribution (*p*-value > 0.05), the results in Table 6 were obtained. For example, Figure 7 shows the empirical cumulative distribution functions of the CD46 and CD58 genetic variables.

### 4.2. Fuzzy System Results

At the fuzzification stage, the linguistic values were computed regarding the numerical values of each input variable. For example, Table 7 shows the linguistic values obtained from $\mu_{A_{1}}$ (age) and $\mu_{A_{2}}$ (initial EDSS) membership functions for all samples.

**Figure 8:**
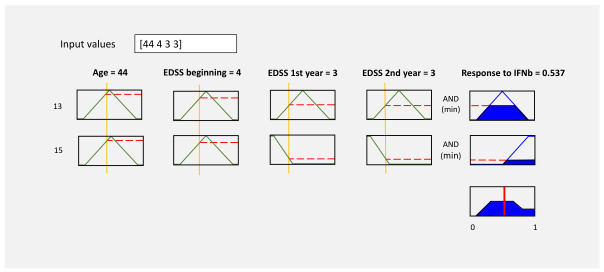
Rule inference graph of the Fuzzy Logic Designer application, describing the results of combining the inference values of the 13 and 15 fuzzy rules. The blue shading area represents the computed inference degree by the inference engine.

At the approximate reasoning stage, each fuzzy rule (knowledge base) was evaluated using the membership values obtained from fuzzification. For example, considering the input values for sample ‘7’ (age: ‘44’; initial EDSS: ‘4’, EDSS after 1 year: ‘3’; EDSS after 2 years: ‘3’), the inference engine results are shown in Table 8. In this case, only two rules had a non-zero inference result. Figure 8 shows the evaluation graph for 13 and 15 fuzzy rules.

**Figure 9:**
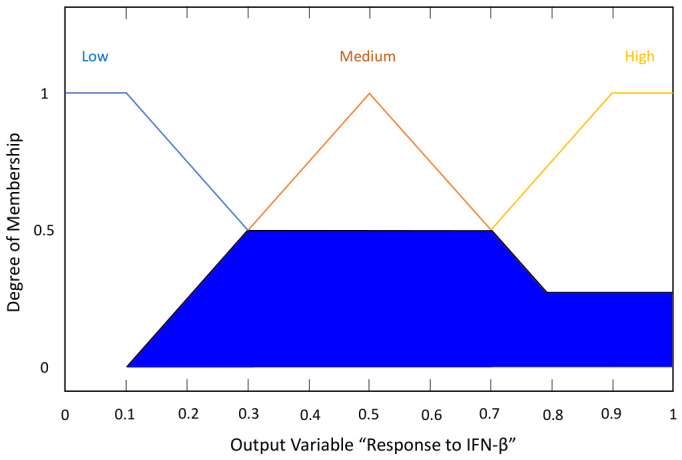
Graph of inference results for sample 7, which displays the values of the linguistic labels: low, medium, and high. The blue shading area represents the calculated membership degree of the response to IFN-β-1b.

At the defuzzification stage, the numerical outputs were calculated from the inference result graphs using Equation (5). For example, to obtain the numerical output for sample 7 according to the inference graph in Figure 9, the following calculation was performed:

(13) $$y_{0}=\frac{0.0\times 0.0+0.1\times 0.0+0.2\times 0.25+0.3\times 0.5+\ldots +0.8\times 0.25+0.9\times 0.25+1\times 0.25}{0.0+0.0+0.25+0.5+0.5+0.5+0.5+0.5+0.25+0.25+0.25}$$

(14) $$y_{0}=\frac{1.9025}{3.4}=0.559\approx 0.537$$

Finally, RRMS patients were classified as high, medium, and low responders to IFN-β-1b treatment using three methods: (1) expert neurology opinion, (2) fuzzy system, and (3) conventional hierarchical clustering model. Table 9 shows the comparison results. The numerical data from Table 1 were also used to train the hierarchical clustering model ($n_{clusters}=3$).

Based on previous results, 80% of patients were properly labeled using the fuzzy system, while 64% were properly labeled using hierarchical clustering, in contrast to expert opinion.

### 4.3. Hyperparameter Optimization Results

**Figure 10:**
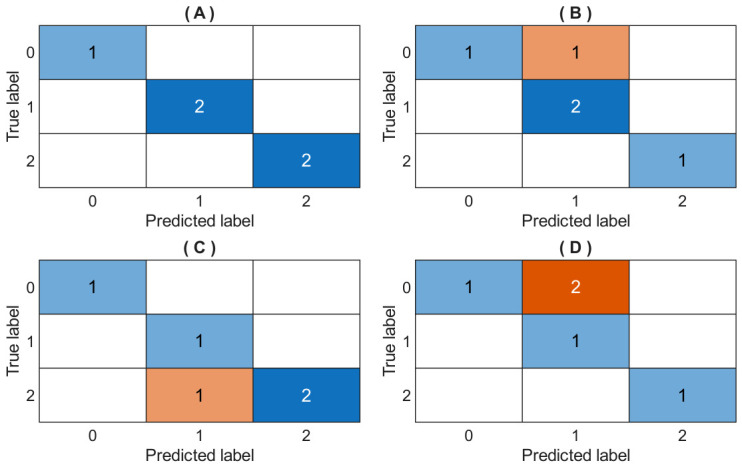
Confusion matrix results for evaluating the ANN model’s prediction performance. (**A**): Fold 1; (**B**): Fold 2; (**C**): Fold 3; (**D**): Fold 4. True and predicted labels represent the outputs as follows: ‘high’ = 0; ‘low’ = 1; ‘medium’ = 2.

In this study, a four-iteration CV method was implemented to evaluate the ANN model’s prediction performance. Table 10 presents the configuration hyperparameter combinations using the GA as an optimizer, and the performance metrics results for each fold are also shown. Figure 10 presents the confusion matrix results to evaluate the accuracy of the ANN model’s classification for four folds.

**Figure 11:**
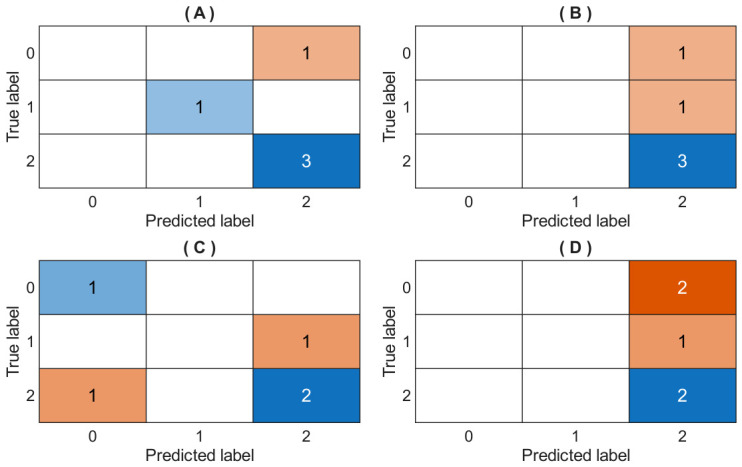
Confusion matrix results for evaluating the MLP model’s prediction performance. (**A**): Fold 1; (**B**): Fold 2; (**C**): Fold 3; (**D**): Fold 4. True and predicted labels represent the outputs as follows: ‘high’ = 0; ‘low’ = 1; ‘medium’ = 2.

**Figure 12:**
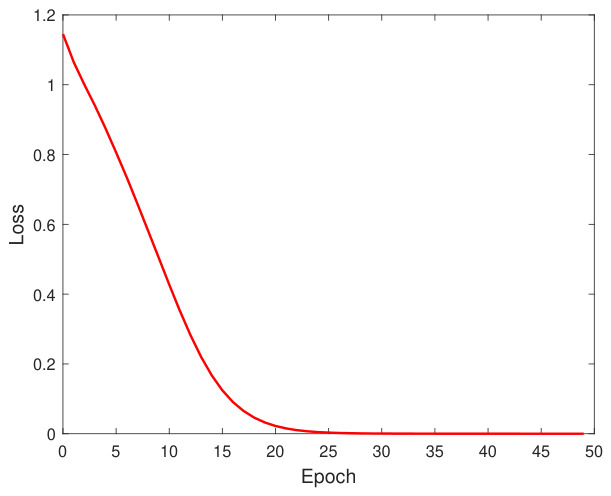
Loss function behavior of the ANN model training process. The loss converges to its minimum value after epoch 25.

Table 11 shows the hyperparameter tuning of the MLP model using the conventional technique GridSearchCV, and the performance metrics results are also shown for each fold. Figure 11 shows the confusion matrix results for evaluating the accuracy of the MLP model’s classification for four folds. Finally, Figure 12 shows the loss function of the ANN model’s training process over 50 epochs (generations).

## 5. Discussion

MS is an autoimmune inflammatory disease of the CNS impacting nearly 3 million people worldwide. Current disease-modifying therapies, such as glatiramer acetate and IFN-β, focus on delaying disease progression, treating sensitive attacks, and improving symptoms [64]. Although these therapies are effective, mainly in the early phases of the disease, some MS patients do not respond or respond only partially, sometimes due to individual genetic variability [18]. Hence, it is important to design new intelligent classification systems to determine the degree of response to MS treatments. ML and DL are subsets of AI that focus on developing computational models to improve specific tasks by making data-based predictions [28]. Over the past decade, the application of ML algorithms has increased across several medical fields [30,31].

In the field of neurology, unsupervised hierarchical clustering methods based on the genome are among the most commonly used to diagnose MS and predict the potential response to IFN-β [39,40]. Although hierarchical clustering methods are easy to implement and the results show that some biomarkers could aid future treatment decisions, these methods rarely provide an efficient solution due to the presence of many arbitrary decisions. Moreover, they do not consider data outliers; i.e., they use only the distance between points to cluster them. Fuzzy logic is another subset of artificial intelligence (AI). A fuzzy system emulates expert reasoning and can handle uncertain and imprecise information to provide efficient solutions. Most studies that have applied fuzzy systems have focused only on the diagnosis of neurological diseases [22,25,26]. Thus, estimating responses to treatments remains a challenge. Hence, in this study, we proposed an AI algorithm that includes a fuzzy system to estimate the possible response to IFN-β-1b treatment in RRMS patients. The fuzzy system is based on fuzzy rules defined in collaboration with a medical specialist. The proposed fuzzy system addresses a limitation of our previous study [47], as the normal distribution of the input variables had not been evaluated. Therefore, the ‘gender’ demographic variable was replaced by the clinical variable ‘Initial EDDS’, as ‘gender’ did not follow a normal distribution relative to other variables. The results shown in Table 9 demonstrate that the fuzzy system achieved greater efficiency in estimating the degree of response to IFN-β1b than hierarchical clustering. Therefore, fuzzy systems are emerging as efficient classification techniques for determining the unknown output labels of any medical database in cases where expert knowledge about the topic is available.

The genetic algorithm (GA) is a population-based metaheuristic algorithm that searches for an optimal solution in the search space. Once the fuzzy system classifies the output labels of the database, a GA is implemented to find the optimal hyperparameter configuration of an ANN model trained with genetic features associated with the response to IFN-β-1b. The performance results in Table 10 and Table 11 indicate that an artificial neural network (ANN) model optimized by a GA can be a useful tool for obtaining reliable predictions on whether MS patients are potential candidates to be treated with this drug. Moreover, the use of the k-iteration CV method to evaluate the performance of the ANN model helped in obtaining a balanced bias–variance rate. Thus, the GA as a hyperparameter optimizer outperforms conventional hyperparameter tuning methods.

In summary, ML and DL algorithms are useful alternatives in the field of bioinformatics because they can identify natural patterns in data. These algorithms have been implemented to improve MS diagnosis [32,33] and aid specialists in predicting the response to drugs in MS patients [35]. Specifically, Table 12 presents a comparison of the performance results of Tao et al. [43] and the results obtained in this study.

However, some difficulties must be overcome, such as acquiring new databases where representative variables are available, including genetic biomarkers, as people in different countries have different demographic and clinical characteristics. Although the sample size (n = 25) used in this study was apparently small, it was compensated by the large number of genes evaluated (1625 expression data = 25 patients × 13 biomarkers × 5 doses of IFN-β-1b). Thus, it is necessary to obtain databases with larger sample sizes to guarantee the satisfactory generalization performance of learning models designed to predict responses to MS treatments. On the other hand, during the design stage of the fuzzy system, there was another issue: the limited input capacity of the Fuzzy Logic Designer application. If more than four or five inputs are entered into the system, processing becomes very slow. Thus, more robust software or devices are needed to manage large amounts of data.

## 6. Conclusions

The proposed AI algorithm for assessing whether IFN-β is suitable for an MS patient is based on a fuzzy system developed in collaboration with a medical specialist and a metaheuristic algorithm for hyperparameter optimization. AI enables the construction of a bridge between a set of variables that may or may not be strongly correlated with the disease. According to the performed analysis, it is possible to estimate whether a patient will have a high, medium, or low response to IFN-β treatment.

The results obtained in this study indicate a promising path for determining better treatment for MS patients if a nonlabeled database is used. Managing uncertainty is one of the challenges in medical decision-making. AI-based predictive systems reduce uncertainty and improve reliability, thereby increasing precision and, consequently, confidence in decision-making about the most effective dose and combination of drugs for MS.

The potential implementation of the proposed AI algorithm as a tool to support medical specialists’ decisions depends on acceptance by the medical authorities of a given clinic or hospital. The first step would be to contact a well-known neurology specialist and present this research and its results regarding the use of AI methods for estimating MS treatment response. The second step would be to acquire sufficient funding or aid from medical institutions to launch an experiment involving blood serum laboratory tests of MS patients who have been treated with a specific drug in recent years. The blood test results would greatly assist in analyzing specific gene expression profiles in the geographic region where the experiment is carried out.

Future studies will be aimed at designing new intelligent systems to improve decision-making for other diseases using different data types: genetic and clinical variables; digital images from medical machines, such as magnetic resonance imaging (MRI); and digital images from special cameras or other devices.

## Figures and Tables

**Table 1:** Demographic and clinical features of RRMS patients treated with IFN-β-1b for two years.

Sample	Age	Initial EDSS ^1^	EDSS ^1^ After a Year	EDSS ^1^ After Two Years
1	63	4	4.5	5.5
2	45	1	1	1
3	25	1	1	1
4	27	4	4	3.5
5	51	3	2.5	2.5
6	41	2	2	4.5
7	44	4	3	3
8	30	1.5	2	2
9	26	4	3.5	3.5
10	42	1	1	1
11	29	2	2	2.5
12	28	1.5	1	2.5
13	48	1	1	1
14	47	3.5	3.5	3
15	42	2	2.5	3
16	50	3.5	3.5	3.5
17	37	1.5	3.0	4.5
18	43	2	1.5	2
19	54	3	2	2
20	40	1	1	1
21	48	2	2	2
22	38	2	3.5	3
23	18	1.5	1.5	2
24	24	1	1	1
25	38	1	1	1

^1^ Expanded Disability Status Scale (EDSS).

**Table 2:** Description of the linguistic variables (demographic, clinical features, and the response to IFN-β-1b).

Membership Function	Fuzzy Set	Universe of Discourse	Labels
$\mu_{A_{1}}$ (Age)	$A_{1}$	$X_{1}$: [0 a 100] years	Child, Adult, Elderly
$\mu_{A_{2}}$ (Initial EDSS)	$A_{2}$	$X_{2}$: [0 a 10] units	Low, Medium, High
$\mu_{A_{3}}$ (EDSS After a Year)	$A_{3}$	$X_{3}$: [0 a 10] units	Low, Medium, High
$\mu_{A_{4}}$ (EDSS After Two Years)	$A_{4}$	$X_{4}$: [0 a 10] units	Low, Medium, High
$\mu_{B}$ (Response to IFN-β1b)	B(y)	Y: [0 a 1] units	Low, Medium, High

**Table 3:** Description of specific fuzzy rules to determine the influence of input variables on the response to IFN-β-1b treatment.

#	Fuzzy Rule
10	If the age is Child Label and the initial EDSS is Low Label, and the EDSS after one year is Medium Label, and the EDSS after two years is High Label, then the response to IFN-β-1b is Low Label.
11	If the age is Child Label and the initial EDSS is Medium Label and the EDSS after one year is High Label and the EDSS after two years is High Label, then the response to IFN-β-1b is Low Label.
12	If the age is Adult Label and the initial EDSS is Low Label, and the EDSS after one year is Low Label, and the EDSS after two years is Low Label, then the response to the IFN-β-1b is Medium Label.
13	If the age is Adult Label and the initial EDSS is Medium Label, and the EDSS after one year is Medium Label, and the EDSS after two years is Medium Label, then the response to the IFN-β-1b is Medium Label.
14	If the age is Adult Label and the initial EDSS is High Label, and the EDSS after one year is High Label, and the EDSS after two years is High Label, then the response to the IFN-β-1b is Medium Label.
15	If the age is Adult Label and the initial EDSS is Medium Label, and the EDSS after one year is Low Label, and the EDSS after two years is Low Label, then the response to the IFN-β-1b is High Label.

**Table 4:** Pseudocode of the implemented GA for hyperparameter optimization.

Initialization	
	Select initial population (number of networks in each generation), N = 10
	Describe the target function f(X), representing accuracy
	Encode the population as chromosomes (bit strings)
	Compute the fitness values (accuracy) of the whole population
	Define the ending condition
	Choose the maximum number of iterations (generations or number of times to evolve the population), $Max_{Iter}=50$
	iter = 1
while (iter ≤ $Max_{Iter}$)	
	Selection: Select parents for breeding, random_select = 0.1
	Crossover: Apply crossover to parents to generate offspring, crossover_chance = 0.4
	Mutation: Apply mutation to selected chromosomes, mutate_chance = 0.2
	Compute the fitness values of the population
	Select members for the next generation according to fitness values
	If the ending condition is met, exit; otherwise, continue (iter = iter + 1)
end while	

**Table 5:** Statistical results of the Kolmogorov–Smirnov test of demographic and clinical variables. ‘Gender’ variable was excluded because it follows a different distribution.

Pairs of Variables	Statistics-*D*	Value-*p*	Distribution
Gender/Age	0.6	0.0001625	Different
Age/Initial EDSS	0.32	0.1557	Normal
Initial EDSS/EDSS After a Year	0.32	0.1557	Normal
EDSS After a Year/EDSS After Two Years	0.28	0.2850	Normal

**Table 6:** Statistical results of the KS test on the genetic variables (first dose).

Pair of Variables	Statistic-*D*	Value-*p*	Distribution
IL-2/IFN-γ	0.12	0.9955	Normal
IFN-γ/TNF-α	0.16	0.9149	Normal
TNF-α/IL-4	0.12	0.9955	Normal
IL-4/IL-10	0.16	0.9149	Normal
IL-10/TGF-β	0.12	0.9955	Normal
TGF-β/CD46	0.24	0.4754	Normal
CD46/CD58	0.2	0.7102	Normal
CD58/FHIT	0.16	0.9149	Normal
FHIT/IFR5	0.16	0.9149	Normal
IFR5/GAPVD1	0.16	0.9149	Normal
GAPVD1/GRM3	0.12	0.9955	Normal
GRM3/GRIK2	0.16	0.9149	Normal

**Table 7:** Fuzzification results regarding the labels of age and initial EDSS input variables.

Sample	$\mu_{\mathrm{Child}}$	$\mu_{\mathrm{Adult}}$	$\mu_{\mathrm{Elderly}}$	$\mu_{\mathrm{Low}}$	$\mu_{\mathrm{Medium}}$	$\mu_{\mathrm{High}}$
1	0.0	0.687	0.015	0.25	0.75	0.0
2	0.0	0.88	0.0	1.0	0.0	0.0
3	0.375	0.4	0.0	1.0	0.0	0.0
4	0.315	0.448	0.0	0.25	0.75	0.0
5	0.0	0.975	0.0	0.5	0.5	0.0
6	0.0	0.784	0.0	0.75	0.25	0.0
7	0.0	0.856	0.0	0.25	0.75	0.0
8	0.225	0.479	0.0	0.875	0.125	0.0
9	0.345	0.424	0.0	0.25	0.75	0.0
10	0	0.808	0.0	1.0	0.0	0.0
11	0.255	0.496	0.0	0.75	0.25	0.0
12	0.258	0.472	0.0	0.875	0.125	0.0
13	0.0	0.952	0.0	1.0	0.0	0.0
14	0.0	0.928	0.0	0.375	0.625	0.0
15	0.0	0.808	0.0	0.75	0.25	0.0
16	0.0	1.0	0.0	0.375	0.625	0.0
17	0.015	0.688	0.0	0.875	0.125	0.0
18	0.0	0.832	0.0	0.75	0.25	0.0
19	0.0	0.903	0.0	0.5	0.5	0.0
20	0.0	0.76	0.0	1.0	0.0	0.0
21	0.0	0.952	0.0	0.75	0.25	0.0
22	0.0	0.712	0.0	0.75	0.25	0.0
23	0.585	0.232	0.0	0.875	0.125	0.0
24	0.405	0.376	0.0	1.0	0.0	0.0
25	0.0	0.712	0.0	1.0	0.0	0.0

**Table 8:** Inference results from 13 and 15 fuzzy rules evaluation (sample ‘7’).

#	Rule	Inference Engine
13	If the age is Adult Label, and the initial EDSS is Medium Label, and the EDSS after one year is Medium Label, and the EDSS after two years is Medium Label, then the IFN-β-1b response is Medium Label.	min(0.856, 0.75, 0.525, 0.5) = 0.5
15	If the age is Adult Label, and the initial EDSS is Medium Label, and the EDSS after one year is Low Label, and the EDSS after two years is Low Label, then the response to IFN-β-1b is High Label.	min(0.856, 0.75, 0.225, 0.5) = 0.225

**Table 9:** Classification of the response to IFN-β-1b. In the case of the fuzzy system, numerical values (resulting from defuzzification) less than 0.5 are regarded as low responder (LR), those equal to 0.5 as medium responder (MR), and those greater than 0.5 as high responder (HR).

Sample	Expert Opinion	Fuzzy System (Defuzzification)	Hierarchical Clustering
1	LR	0.5 ⇒ MR	HR
2	MR	0.5 ⇒ MR	MR
3	MR	0.5 ⇒ MR	MR
4	MR	0.5 ⇒ MR	HR
5	MR	0.537 ⇒ HR	LR
6	LR	0.433 ⇒ LR	LR
7	HR	0.537 ⇒ HR	HR
8	MR	0.473 ⇒ LR	MR
9	MR	0.513 ⇒ HR	HR
10	MR	0.5 ⇒ MR	MR
11	MR	0.5 ⇒ MR	MR
12	LR	0.473 ⇒ LR	MR
13	MR	0.5 ⇒ MR	MR
14	MR	0.5 ⇒ MR	HR
15	LR	0.443 ⇒ LR	LR
16	MR	0.5 ⇒ MR	HR
17	LR	0.247 ⇒ LR	LR
18	MR	0.527 ⇒ HR	LR
19	HR	0.595 ⇒ HR	LR
20	MR	0.5 ⇒ MR	MR
21	MR	0.5 ⇒ MR	LR
22	LR	0.363 ⇒ LR	LR
23	MR	0.5 ⇒ MR	MR
24	MR	0.5 ⇒ MR	MR
25	MR	0.5 ⇒ MR	MR

**Table 10:** Configuration hyperparameters optimized by GA and performance metrics results using four-iteration CV (four models with performance estimations).

Fold	Hidden Neurons	Hidden Layers	Activation Function	Optimizer	Accuracy	Precision	Recall
1	64	4	‘tanh’	‘adamax’ ^1^	1.0	1.0	1.0
2	128	4	‘tanh’	‘adamax’	0.8	0.88	0.83
3	64	4	‘tanh’	‘adagrad’ ^2^	0.8	0.83	0.88
4	64	4	‘tanh’	‘adamax’	0.6	0.77	0.77

^1^ Adaptive Moment Estimation (Adam) variant that performs parameter updates by computing the gradient across the entire dataset. ^2^ Adam variant well-founded in the infinite norm is a first-order gradient-based optimization method.

**Table 11:** Configuration hyperparameter tuning of the MLP model using GridSearchCV and performance metrics results using four-iteration CV (four models of the best selected hyperparameters).

Fold	Hidden Neurons	Hidden Layers	Activation Function	Solver	Accuracy	Precision	Recall
1	64	1	‘logistic’	‘sgd’ ^1^	0.8	0.65	0.8
2	128	1	‘logistic’	‘sgd’	0.6	0.36	0.6
3	256	4	‘logistic’	‘sgd’	0.6	0.5	0.6
4	512	3	‘logistic’	‘sgd’	0.4	0.16	0.4

^1^ Stochastic gradient descent.

**Table 12:** Performance results comparison of Tao et al.’s study [43] and our research.

Author	Prediction	Method	Accuracy
[43]	Response to IFN-β-1b	DCE ^1^/SVM ^2^	0.8095
Our work	Response to IFN-β-1b	FS ^3^/GA ^4^/ANN ^5^	0.8–1.0

^1^ Differentially correlated genes; ^2^ support vector machine; ^3^ fuzzy system; ^4^ genetic algorithm; ^5^ artificial neural network.

## Data Availability

The collected gene expression data are available at https://github.com/eponcedeleon13-max/GSE24427_Gene_Expression_Data (accessed on 15 October 2025).

## Abbreviations

The following abbreviations are used in this manuscript:

ADAs	Anti-drug antibodies;
Adam	Adaptive moment estimation;
AI	Artificial intelligence;
ANN	Artificial neural network;
BBB	Blood–brain barrier;
CM	Confusion matrix;
CNS	Central nervous system;
COG	Center of gravity;
CV	Cross-validation;
DCE	Differentially correlated gene;
DEG	Differentially expressed gene;
DL	Deep learning;
DMP	Differentially methylated position;
DNA	Deoxyribonucleic acid;
DT	Decision tree;
EDSS	Expanded disability status scale;
FS	Fuzzy system;
GA	Genetic algorithm;
GBTM	Group-based trajectory modeling;
GEO	Gene Expression Omnibus;
IFN	Interferon;
KN	K-neighbor;
KS	Kolmogorov–Smirnov;
MAFS	Mamdani–Assilan fuzzy system;
ML	Machine learning;
MLP	Multilayer perceptron;
MRI	Magnetic resonance imaging;
MS	Multiple sclerosis;
NAWM	Normal-appearing white matter;
PCA	Principal component analysis;
PPMS	Primary progressive multiple sclerosis;
RF	Random forest;
RNA	Ribonucleic acid;
RRMS	Relapsing–remitting multiple sclerosis;
SGD	Stochastic gradient descent;
SPMS	Secondary progressive multiple sclerosis;
SVM	Support vector machine;
UCSC	Uncorrelated shrunken centroid.

## Appendix A

**Table A1:** Definition of fuzzy rules to determine the influence of input variables on the response to IFN-β-1b treatment (first part).

#	Fuzzy Rule
1	If the age is Child Label, and the initial EDSS is Low Label, and the EDSS after one year is Low Label, and the EDSS after two years is Low Label, then the response to IFN-β-1b is Medium Label.
2	If the age is Child Label, and the initial EDSS is Medium Label, and the EDSS after one year is Medium Label, and the EDSS after two years is Medium Label, then the response to IFN-β-1b is Medium Label.
3	If the age is Child Label, and the initial EDSS is High Label, and the EDSS after one year is High Label, and the EDSS after two years is High Label, then the response to IFN-β-1b is Medium Label.
4	If the age is Child Label, and the initial EDSS is Medium Label, and the EDSS after one year is Low Label, and the EDSS after two years is Low Label, then the response to IFN-β-1b is High.
5	If the age is Child Label, and the initial EDSS is High Label, and the EDSS after one year is Medium Label, and the EDSS after two years is Medium Label, then the response to the IFN-β-1b is High Label.
6	If the age is Child Label and the initial EDSS is High Label, and the EDSS after one year is Low Label, and the EDSS after two years is Low Label, then the response to the IFN-β-1b is High Label.
7	If the age is Child Label, and the initial EDSS is High Label, and the EDSS after one year is Medium Label, and the EDSS after two years is Low Label, then the response to the IFN-β-1b is High Label.
8	If the age is Child Label, and the initial EDSS is Low Label, and the EDSS after one year is Medium Label, and the EDSS after two years is Medium Label, then the response to the IFN-β-1b is Low Label.
9	If the age is Child Label, and the initial EDSS is Low Label, and the EDSS after one year is High Label, and the EDSS after two years is High Label, then the response to IFN-β-1b is Low Label.
10	If the age is Child Label, and the initial EDSS is Low Label, and the EDSS after one year is Medium Label, and the EDSS after two years is High Label, then the response to IFN-β-1b is Low Label.
11	If the age is Child Label, and the initial EDSS is Medium Label, and the EDSS after one year is High Label, and the EDSS after two years is High Label, then the response to IFN-β-1b is Low Label.
12	If the age is Adult Label, and the initial EDSS is Low Label, and the EDSS after one year is Low Label, and the EDSS after two years is Low Label, then the response to the IFN-β-1b is Medium Label.
13	If the age is Adult Label, and the initial EDSS is Medium Label, and the EDSS after one year is Medium Label, and the EDSS after two years is Medium Label, then the response to the IFN-β-1b is Medium Label.
14	If the age is Adult Label, and the initial EDSS is High Label, and the EDSS after one year is High Label, and the EDSS after two years is High Label, then the response to the IFN-β-1b is Medium Label.
15	If the age is Adult Label, and the initial EDSS is Medium Label, and the EDSS after one year is Low Label, and the EDSS after two years is Low Label, then the response to the IFN-β-1b is High Label.
16	If the age is Adult Label, and the initial EDSS is High Label, and the EDSS after one year is Medium Label, and the EDSS after two years is Medium Label, then the response to IFN-β-1b is High Label.
17	If the age is Adult Label, and the initial EDSS is High Label, and the EDSS after one year is Low Label, and the EDSS after two years is Low Label, then the response to the IFN-β-1b is High Label.

**Table A2:** Definition of fuzzy rules to determine the influence of input variables on the response to IFN-β-1b treatment (second part).

#	Fuzzy Rule
18	If the age is Adult Label, and the initial EDSS is High Label, and the EDSS after one year is Medium Label, and the EDSS after two years is Low Label, then the response to the IFN-β-1b is High Label.
19	If the age is Adult Label, and the initial EDSS is Low Label, and the EDSS after one year is Medium Label, and the EDSS after two years is Medium Label, then the response to the IFN-β-1b is Low Label.
20	If the age is Adult Label, and the initial EDSS is Low Label, and the EDSS after one year is High Label, and the EDSS after two years is High Label, then the response to the IFN-β-1b is Low Label.
21	If the age is Adult Label, and the initial EDSS is Low Label, the EDSS after one year is Medium Label, and the EDSS after two years is High Label, then the response to the IFN-β-1b is Low Label.
22	If the age is Adult Label, and the initial EDSS is Medium Label, the EDSS after one year is High Label, and the EDSS after two years is High Label, then the response to the IFN-β-1b test is Low Label.
23	If the age is Elderly Label, and the initial EDSS is Low Label, and the EDSS after one year is Low Label, and the EDSS after two years is Low Label, then the response to the IFN-β-1b is Medium Label.
24	If the age is Elderly Label, and the initial EDSS is Medium Label, and the EDSS after one year is Medium Label, and the EDSS after two years is Medium Label, then the response to the IFN-β-1b is Medium Label.
25	If the age is Elderly Label, and the initial EDSS is High Label, and the EDSS after one year is High Label, and the EDSS after two years is High Label, then the response to IFN-β-1b is Medium Label.
26	If the age is Elderly Label, and the initial EDSS is Medium Label, and the EDSS after one year is Low Label, and the EDSS after two years is Low Label, then the response to IFN-β-1b is High Label.
27	If the age is Elderly Label, and the initial EDSS is High Label, and the EDSS after one year is Medium Label, and the EDSS after two years is Medium Label, then the response to IFN-β-1b is High Label.
28	If the age is Elderly Label, and the initial EDSS is High Label, and the EDSS after one year is Low Label, and the EDSS after two years is Low Label, then the response to IFN-β-1b is High Label.
29	If the age is Elderly Label, and the initial EDSS is High Label, and the EDSS after one year is Medium Label, and the EDSS after two years is Low Label, then the response to IFN-β-1b is High Label.
30	If the age is Elderly Label, and the initial EDSS is Low Label, and the EDSS after one year is Medium Label, and the EDSS after two years is Medium Label, then the response to IFN-β-1b is Low Label.
31	If the age is Elderly Label, and the initial EDSS is Low Label, and the EDSS after one year is High Label, and the EDSS after two years is High Label, then the response to IFN-β-1b is Low Label.
32	If the age is Elderly Label, and the initial EDSS is Low Label, and the EDSS after one year is Medium Label, and the EDSS after two years is High Label, then the response to IFN-β-1b is Low Label.
33	If the age is Elderly Label, and the initial EDSS is Medium Label, and the EDSS after one year is High Label, and the EDSS after two years is High Label, then the response to IFN-β-1b is Low Label.

## References

[1] Rodríguez Murúa S., Farez M.F., Quintana F.J. (2022). The immune response in multiple sclerosis. Annu. Rev. Pathol. Mech. Dis..

[2] Aslam N., Khan I.U., Bashamakh A., Alghool F.A., Aboulnour M., Alsuwayan N.M., Alturaif R.K., Brahimi S., Aljameel S.S., Al Ghamdi K. (2022). Multiple Sclerosis Diagnosis Using Machine Learning and Deep Learning: Challenges and Opportunities. Sensors.

[3] Margarit B.P., Monteiro G.C., Herán I.S., Delgado F.R., Izquierdo A.Y. (2019). Esclerosis múltiple. Med. Accredit. Contin. Med. Educ. Program.

[4] Inojosa H., Proschmann U., Akgün K., Ziemssen T. (2021). A focus on secondary progressive multiple sclerosis (SPMS): Challenges in diagnosis and definition. J. Neurol..

[5] Ghasemi N., Razavi S., Nikzad E. (2016). Multiple sclerosis: Pathogenesis, symptoms, diagnoses and cell-based therapy. Cell J..

[6] Tarlinton R.E., Martynova E., Rizvanov A.A., Khaiboullina S., Verma S. (2020). Role of viruses in the pathogenesis of multiple sclerosis. Viruses.

[7] Zarghami A., Li Y., Claflin S.B., van der Mei I., Taylor B.V. (2021). Role of environmental factors in multiple sclerosis. Expert Rev. Neurother..

[8] Dominguez-Mozo M.I., Perez-Perez S., Villarrubia N., Costa-Frossard L., Fernandez-Velasco J.I., Ortega-Madueño I., Alvarez-Lafuente R. (2021). Herpesvirus antibodies, vitamin d and short-chain fatty acids: Their correlation with cell subsets in multiple sclerosis patients and healthy controls. Cells.

[9] Lorscheider J., Benkert P., Lienert C., Hänni P., Derfuss T., Kuhle J., Yaldizli Ö. (2018). Comparative analysis of natalizumab versus fingolimod as second-line treatment in relapsing–remitting multiple sclerosis. Mult. Scler. J..

[10] Mirandola S.R., Hallal D.E., Farias A.S., Oliveira E.C., Brandão C.O., Ruocco H.H., Santos L.M. (2009). Interferon-beta modifies the peripheral blood cell cytokine secretion in patients with multiple sclerosis. Int. Immunopharmacol..

[11] Kay M., Hojati Z., Dehghanian F. (2013). The molecular study of IFN-*β* pleiotropic roles in MS treatment. Iran. J. Neurol..

[12] Hočevar K., Ristić S., Peterlin B. (2019). Pharmacogenomics of multiple sclerosis: A systematic review. Front. Neurol..

[13] Li L., Olcer M., Wang Z., Song Y., Ke J., Feng X., Reder A.T. (2025). IFN-*β* therapy rescues dysregulated IFN-stimulated proteins, serum cytokines, and neurotrophic factors in multiple sclerosis: Multiplex analysis of short-term and long-term IFN responses. PLoS ONE.

[14] Ganji A., Monfared M.E., Shapoori S., Nourbakhsh P., Ghazavi A., Ghasami K., Mosayebi G. (2020). Effects of interferon and glatiramer acetate on cytokine patterns in multiple sclerosis patients. Cytokine.

[15] Martínez-Aguilar L., Pérez-Ramírez C., del Mar Maldonado-Montoro M., Carrasco-Campos M.I., Membrive-Jiménez C., Martínez-Martínez F., Jiménez-Morales A. (2015). Effect of genetic polymorphisms on therapeutic response in multiple sclerosis relapsing-remitting patients treated with interferon-beta. Mutat. Res..

[16] Cohan S.L., Hendin B.A., Reder A.T., Smoot K., Avila R., Mendoza J.P., Weinstock-Guttman B. (2021). Interferons and multiple sclerosis: Lessons from 25 years of clinical and real-world experience with intramuscular interferon beta-1a (Avonex). CNS Drugs.

[17] Bustamante M.F., Morcillo-Suárez C., Malhotra S., Rio J., Leyva L., Fernández O., Comabella M. (2015). Pharmacogenomic study in patients with multiple sclerosis: Responders and nonresponders to IFN-*β*. Neuroimmunol. Neuroinflamm..

[18] Río J., Nos C., Tintoré M., Téllez N., Galán I., Pelayo R., Montalban X. (2006). Defining the response to interferon-*β* in relapsing-remitting multiple sclerosis patients. Ann. Neurol. Off. J. Am. Neurol. Assoc. Child Neurol. Soc..

[19] Vela M.E.F.V., Gutiérrez-Aguilar R. (2020). Ciencias “ómicas”, ¿cómo ayudan a las ciencias de la salud?. Rev. Digit. Univ..

[20] Karlik B. (2013). Soft computing methods in bioinformatics: A comprehensive review. Math. Comput. Appl..

[21] Saibene A., Assale M., Giltri M. (2021). Expert systems: Definitions, advantages and issues in medical field applications. Expert Syst. Appl..

[22] Sadoughi F., Arani L.A. (2022). Intelligent computer systems for multiple sclerosis diagnosis. Augmenting Neurological Disorder Prediction and Rehabilitation Using Artificial Intelligence.

[23] Ayangbekun O.J., Jimoh Ibrahim A. (2015). Fuzzy logic application to brain diseases diagnosis. J. Emerg. Trends Comput. Inf. Sci..

[24] Hosseini A., Asadi F., Arani L.A. (2020). Development of a knowledge-based clinical decision support system for multiple sclerosis diagnosis. J. Med. Life.

[25] Matinfar F., Golpaygani A.T. (2022). A fuzzy expert system for early diagnosis of multiple sclerosis. J. Biomed. Phys. Eng..

[26] Chen J., Gustientiedina G. (2024). Implementation of Fuzzy Expert System to Detect Parkinson’s Disease Based on Mobile. J. Appl. Bus. Technol..

[27] Law M.T., Traboulsee A.L., Li D.K., Carruthers R.L., Freedman M.S., Kolind S.H., Tam R. (2019). Machine learning in secondary progressive multiple sclerosis: An improved predictive model for short-term disability progression. Mult. Scler. J. Exp. Transl. Clin..

[28] Meskó B., Görög M. (2020). A short guide for medical professionals in the era of artificial intelligence. NPJ Digit. Med..

[29] Cavaliere C., Vilades E., Alonso-Rodríguez M.C., Rodrigo M.J., Pablo L.E., Miguel J.M., López-Guillén E., Morla E.M.S., Boquete L., Garcia-Martin E. (2019). Computer-Aided Diagnosis of Multiple Sclerosis Using a Support Vector Machine and Optical Coherence Tomography Features. Sensors.

[30] Briganti G., Le Moine O. (2020). Artificial intelligence in medicine: Today and tomorrow. Front. Med..

[31] Haug C.J., Drazen J.M. (2023). Artificial intelligence and machine learning in clinical medicine. N. Engl. J. Med..

[32] Goyal M., Khanna D., Rana P.S., Khaibullin T., Martynova E., Rizvanov A.A., Baranwal M. (2019). Computational intelligence technique for prediction of multiple sclerosis based on serum cytokines. Front. Neurol..

[33] Chen X., Hou H., Qiao H., Fan H., Zhao T., Dong M. (2021). Identification of blood-derived candidate gene markers and a new 7-gene diagnostic model for multiple sclerosis. Biol. Res..

[34] Casalino G., Castellano G., Consiglio A., Nuzziello N., Vessio G. (2023). MicroRNA expression classification for pediatric multiple sclerosis identification. J. Ambient Intell. Humaniz. Comput..

[35] Fagone P., Mazzon E., Mammana S., Di Marco R., Spinasanta F., Basile M.S., Mangano K. (2019). Identification of CD4+ T cell biomarkers for predicting the response of patients with relapsing-remitting multiple sclerosis to natalizumab treatment. Mol. Med. Rep..

[36] Ebrahimkhani S., Beadnall H.N., Wang C., Suter C.M., Barnett M.H., Buckland M.E., Vafaee F. (2020). Serum exosome microRNAs predict multiple sclerosis disease activity after fingolimod treatment. Mol. Neurobiol..

[37] Giordani P., Ferraro M.B., Martella F. (2020). Non-Hierarchical Clustering. An Introduction to Clustering with R.

[38] Vichi M., Cavicchia C., Groenen P.J. (2022). Hierarchical means clustering. J. Classif..

[39] Gurevich M., Miron G., Falb R.Z., Magalashvili D., Dolev M., Stern Y., Achiron A. (2015). Transcriptional response to interferon beta-1a treatment in patients with secondary progressive multiple sclerosis. BMC Neurol..

[40] Coelewij L., Adriani M., Dönnes P., Waddington K.E., Ciurtin C., Havrdova E.K., ABIRISK Consortium (2022). Patients with multiple sclerosis who develop immunogenicity to interferon-beta have distinct transcriptomic and proteomic signatures prior to treatment which are associated with disease severity. Clin. Immunol..

[41] Mésidor M., Rousseau M.C., Duquette P., Sylvestre M.P. (2021). Classification and visualization of longitudinal patterns of medication dose: An application to interferon-beta-1a and amitriptyline in patients with multiple sclerosis. Pharmacoepidemiol. Drug Saf..

[42] Kular L., Ewing E., Needhamsen M., Pahlevan Kakhki M., Covacu R., Gomez-Cabrero D., Jagodic M. (2022). DNA methylation changes in glial cells of the normal-appearing white matter in Multiple Sclerosis patients. Epigenetics.

[43] Tao J., Wang C., Tian S. (2020). Feature selection based on differentially correlated gene pairs reveals the mechanism of IFN-therapy for multiple sclerosis. Bioinform. Genom..

[44] Madhiarasan M., Deepa S.N. (2020). A novel criterion to select hidden neuron numbers in improved back propagation networks for wind speed forecasting. Appl. Intell..

[45] Yang X.S. (2020). Nature-Inspired Optimization Algorithms.

[46] Robert C. (2021). Statistics and Analysis of Scientific Data.

[47] de Leon-Sanchez E.R.P., Mendiola-Santibañez J.D., Dominguez-Ramirez O.A., Herrera-Navarro A.M., Vazquez-Cervantes A., Jimenez-Hernandez H., Senties-Madrid H. (2023). Fuzzy logic system for classifying multiple sclerosis patients as high, medium, or low responders to interferon-beta. Technologies.

[48] Czabanski R., Jezewski M., Leski J. (2017). Introduction to fuzzy systems. Theory and Applications of Ordered Fuzzy Numbers.

[49] Rutkowska D. (2001). Neuro-Fuzzy Architectures and Hybrid Learning.

[50] Salgado-Gomes-Sagaz F., Zorrilla-Muñoz V., Garcia-Aracil N. (2024). Rehabilitation Technologies by Integrating Exoskeletons, Aquatic Therapy, and Quantum Computing for Enhanced Patient Outcomes. Sensors.

[51] Prokopowicz P., Czerniak J., Mikołajewski D., Apiecionek Ł., Ślezak D. (2017). Theory and Applications of Ordered Fuzzy Numbers: A Tribute to Professor Witold Kosiński.

[52] Ackermann M.R., Blömer J., Kuntze D., Sohler C. (2014). Analysis of agglomerative clustering. Algorithmica.

[53] Mirjalili V., Raschka S. (2020). Python Machine Learning.

[54] Jha A.R., Pillai D.G. (2021). Mastering PyTorch.

[55] Mohammed M., Khan M.B., Bashier E.B.M. (2016). Machine Learning: Algorithms and Applications.

[56] Montolio A., Martín-Gallego A., Cegoñino J., Orduna E., Vilades E., Garcia-Martin E., Del Palomar A.P. (2021). Machine learning in diagnosis and disability prediction of multiple sclerosis using optical coherence tomography. Comput. Biol. Med..

[57] Sarker I.H. (2021). Machine learning: Algorithms, real-world applications and research directions. SN Comput. Sci..

[58] Kotsiantis S.B. (2013). Decision trees: A recent overview. Artif. Intell. Rev..

[59] Stevens E., Antiga L., Viehmann T. (2020). Deep Learning with PyTorch.

[60] Roccetti M., Delnevo G., Casini L., Mirri S. (2021). An alternative approach to dimension reduction for pareto distributed data: A case study. J. Big Data.

[61] Villegas-Mier C.G., Rodriguez-Resendiz J., Álvarez-Alvarado J.M., Jiménez-Hernández H., Odry A. (2022). Optimized random forest for solar radiation prediction using sunshine hours. Micromachines.

[62] Garcia-Villegas E., Lopez-Garcia A., Lopez-Aguilera E. (2023). Genetic Algorithm-Based Grouping Strategy for IEEE 802.11ah Networks. Sensors.

[63] Vasuki A. (2020). Nature-Inspired Optimization Algorithms.

[64] Torkildsen Ø., Myhr K.M., Bø L. (2016). Disease-modifying treatments for multiple sclerosis—A review of approved medications. Eur. J. Neurol..

